# In Vitro Cytotoxic Protective Effect of Alginate-Encapsulated Capsaicin Might Improve Skin Side Effects Associated with the Topical Application of Capsaicin

**DOI:** 10.3390/molecules26051455

**Published:** 2021-03-07

**Authors:** Ariana Hudita, Bianca Galateanu, Marieta Costache, Carolina Negrei, Rodica-Mariana Ion, Lorena Iancu, Octav Ginghina

**Affiliations:** 1 Department of Biochemistry and Molecular Biology, University of Bucharest, 050095 Bucharest, Romania; arianahudita@yahoo.com (A.H.); Bianca.galateanu@bio.unibuc.ro (B.G.); Marieta.costache@bio.unibuc.ro (M.C.); 2 Department of Toxicology, Faculty of Pharmacy, “Carol Davila” University of Medicine and Pharmacy, 020021 Bucharest, Romania; 3 National Institute of Research and Development for Chemistry and Petrochemistry—ICECHIM, 060021 Bucharest, Romania; rodica.ion@icechim.ro (R.-M.I.); lorena.iancu@icechim.ro (L.I.); 4 Department of Surgery, “Sf. Ioan” Clinical Emergency Hospital, 042122 Bucharest, Romania; octavginghina@yahoo.com; 5 Department II, Faculty of Dental Medicine, “Carol Davila” University of Medicine and Pharmacy, 020021 Bucharest, Romania

**Keywords:** capsaicin, alginate encapsulation, neuropathic pain, cytotoxicity, inflammation

## Abstract

Chronic neuropathic pain, particularly peripheral pain, is a cause of great concern for diabetic patients. Current treatments include numerous agents such as capsaicinoids, a known deterrent of neuropathic pain despite the inconvenience associated with local side effects. In this context, the current work aims to elucidate the potential mechanisms involved in cytotoxicity by capsaicin and proposes an efficient formulation of capsaicin in alginate microcapsules, which significantly reduces side effects from capsaicin topical administration. For this, human dermal fibroblast cells were treated with alginate-microencapsulated capsaicin extracts and screened for potential cytotoxic effects produced by the treatment. Cell viability and morphology were examined, as well as oxidative stress status and anti-inflammatory potential. Our results show that the alginate encapsulated formulation of capsaicin exerted lower cytotoxic effects on human dermal fibroblasts as measured by cell viability and reactive oxygen species (ROS) production. Furthermore, the expression profiles of inflammatory cytokines were significantly altered by the treatment as compared with the control culture.

## 1. Introduction

The use of capsaicinoids for the treatment of pain goes as far back as 4000 BC [1]. However, it was only introduced to the Western world in the 15th century, on Columbus’ return to Europe from his successful discovery trip to the Americas, and chilies were one of the wonder products he brought over. First only used as a very attractive spice, the medicinal powers of chilies (and capsaicinoids) were discovered much later in the middle of the 19th century, when their peculiar capacity to alleviate pain by topical application turned them into a very appreciated remedy against burns or itches in the extremities [2]. Also known as “green peppers”, this fruit’s name is rather improper, since they are a member of the genus *Capsicum*. They owe their hot and pungent taste not so much to capsaicin as to piperines, the usual and defining component of the Piperaceae family [3]. From over 20 known major non-endogenous capsaicinoids, most (as much as 90%) naturally occurring ones are capsaicin and dihydrocapsaicin [4]). Others include homodihydrocapsaicin, nordihydrocapsaicin, and homocapsaicin [4].

As a result of continued research, further medicinal properties of capsaicinoids were discovered, and they are currently studied for their potential as analgesics, antioxidants, anticarcinogens [5], and pharmacological agents against obesity [4,6,7,8].

Capsaicin intended for trade purposes is an oily extract, authorized as a cream or patch, which is used in the treatment of chronic pain syndromes as postherpetic neuralgia, musculoskeletal pain, arthrosis, rheumatoid arthritis, rash, psoriasis, bladder conditions (neurogenic bladder), and, last but not least, diabetic neuropathy [9,10]. Capsaicin (*trans*-8-methyl-*N*-vanillyl-6-nonenamide), known as chili pepper fruit, is a natural alkaloid derived from plants of the genus *Capsicum*. Like other vanilloids, capsaicin has a benzene ring and a long hydrophobic carbon tail with a polar amide group. Capsaicin is not water-soluble, and for its solubilization, different alcohols and other organic solvents are used in topical preparations and sprays. In order to keep capsaicin stable for a long time and to increase its solubility, this compound has been encapsulated by different biomaterials, such as alginate, which is recognized for its low toxicity. From a chemical point of view, alginate is a linear copolymer containing blocks of (1, 4)-linked β-d-mannuronate and α-l-guluronate residues. Alginate is useful as a matrix for cell immobilization, as well as for the entrapment of bioactive compounds and drugs. Encapsulated drugs are released from alginate pellets by diffusional processes through pores in the polymeric network [11].

Chronic neuropathic pain, particularly peripheral pain related to diabetes, is a cause for concern for several reasons, including the worsening of pain at night, which causes sleep deprivation and an entire range of related subsequent effects such as fatigue, poor performance, and poor social integration.

Treatment is typically aimed at pain modulation, patient education regarding pain management, and restoration of motor function, all relying on constant and careful glycemic control [11]. The treatments available have been limited by adverse reactions, leading to suboptimal benefit/risk ratio. Furthermore, among the numerous agents researched, there has been renewed interest in finding further means to use the analgesic action of capsaicinoids as a deterrent of neuropathic pain, resulting in the emergence on the market and pharmaceutical development of a variety of capsaicin-containing products. These products include quasi-traditional OTC capsaicin preparations and low (<1.0%) concentration capsaicin. Such efforts were proven necessary by the comparatively unsatisfactory efficacy of existing products, which has been aggravated by poor patient compliance arising from the need for multiple topical applications over extended periods as a result of insufficient effectiveness. Among the solutions proposed and tested, the development of products with increased capsaicin strength, i.e., the capsaicin 8% patch, have shown promising potential to alleviate pain by a single topical application.

In regard to the management of diabetic neuropathic peripheral pain, studies suggest that capsaicin may be effective to a certain degree, but its use is fraught with a frustrating number of limitations. Inconveniences such as an unpleasant burning sensation on initial application, the extended time needed for sufficient depletion of pain, the necessity for multiple applications to maintain analgesic efficacy, problematic effects in case of discontinuation for longer than 24 h, and compelling sustained capsaicin application for substance P replenishing all inflict upon the potential for the widespread use of current capsaicin pharmaceutical formulations, aggravating the need to develop new formulations of enhanced efficacy and limited adverse effects [12,13].

In this context, this work aims to elucidate the potential mechanisms involved in the cytotoxicity of capsaicin and proposes an efficient formulation of capsaicin in alginate microcapsules, which significantly reduces the side effects of the topical administration of capsaicin.

## 2. Results and Discussion

### 2.1. Alginate Microcapsules and Capsaicin Encapsulation

**Figure 1:**
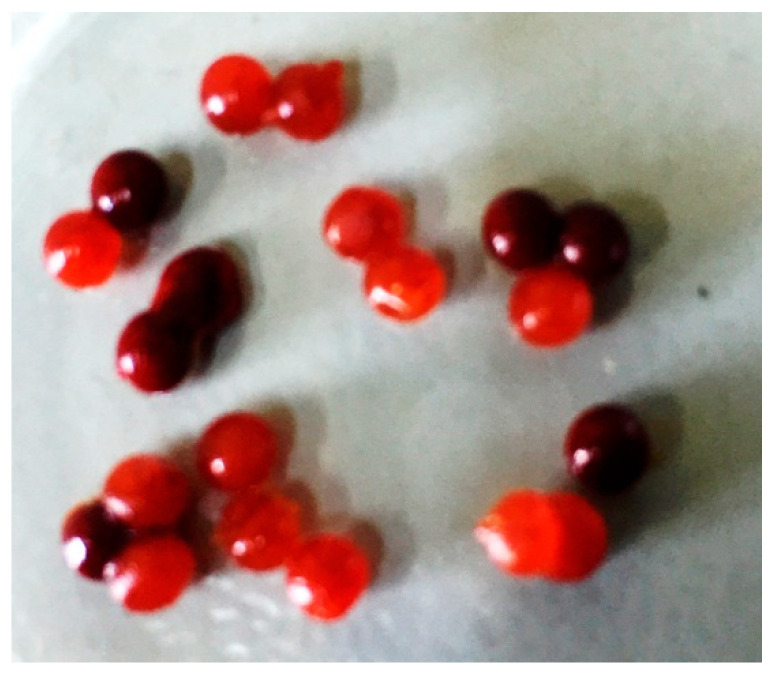
Microscopy image of alginate microcapsules loaded with capsaicin.

The encapsulation efficiency (EE, %) is a variable that allows us to quantify the amount of active principle (capsaicin, in this case) that is encapsulated and can be calculated by analyzing the amount that has not been encapsulated or the amount that is lost during the microparticle consolidation stage. Quantification was carried out by UV-vis. Calcium alginate spherical microcapsules, shown in Figure 1, were prepared by dropping a sodium alginate solution containing a suspension of the gelatin microparticles into a CaCl_2_ solution.

The encapsulation efficiency of capsaicin can be expressed by the Equation (1), presented in the Materials and Methods section. For these experiments, by applying the above-mentioned formula, the obtained encapsulation efficiency was 87%.

### 2.2. In Vitro Cytotoxic Activity of Capsaicin-Loaded Alginate Microcapsules

**Figure 2:**
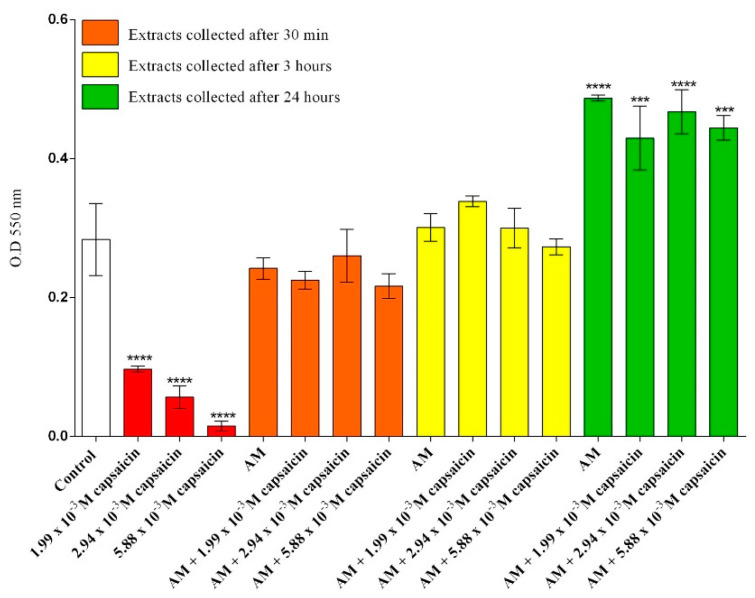
Measurement of cell viability after 24 h of exposure to different treatments as revealed by MTT assay (*** *p* ≤ 0.001 treated vs. control; **** *p* ≤ 0.0001 treated vs. control).

The viability of CCD-1070Sk dermal fibroblasts was investigated after 24 h of treatment with the collected extracts and different concentrations of free capsaicin. Cellular mitochondrial activity was analyzed using the MTT quantitative assay. The obtained data were statistically analyzed using GraphPad Prism software and are graphically represented in Figure 2. The viability of dermal fibroblasts exposed to free capsaicin decreased significantly after 24 h of treatment as compared to that of the untreated samples, regardless of the concentration of capsaicin used. In comparison, extracts of AM (alginate microcapsules) and AM loaded with capsaicin, collected at 30 min and 3 h, did not show any significant effect on CCD-1070Sk cell viability after 24 h of treatment as compared with the untreated samples. Furthermore, CCD-1070Sk cells exposed to capsaicin loaded AM extracts for 24 h had a statistically significant increase in cellular viability for all concentrations of capsaicin, demonstrating that the microencapsulation of capsaicin alleviates its cytotoxicity.

**Figure 3:**
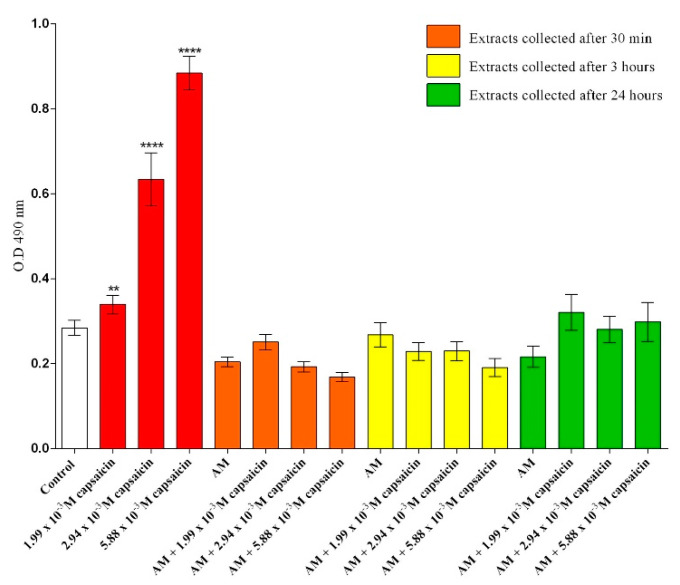
Quantification of LDH release after 24 h of exposure to different experimental conditions (** *p* ≤ 0.01 treated vs. control; **** *p* ≤ 0.0001 treated vs. control).

To further explore the cytotoxicity of the collected extracts and different concentrations of free capsaicin, the release of lactate dehydrogenase (LDH) in culture cell media was measured after 24 h of treatment. As shown in Figure 3, a statistically significant increase in LDH release was observed in response to 24 h exposure of CCD-1070Sk cells to free capsaicin solutions for all screened concentrations, highlighting the cytotoxic potential of free capsaicin on human dermal fibroblasts. Furthermore, the cytotoxic profile of the extracts collected from unloaded AM revealed that the bare micro formulation did not trigger any alteration in the LDH activity after 24 h of treatment. Also, the same cytotoxic profile was noticed for the extracts collected from the capsaicin-loaded AM for all concentrations tested, showing that the microencapsulation of capsaicin represents a strong tool for modulating free capsaicin cytotoxicity.

**Figure 4:**
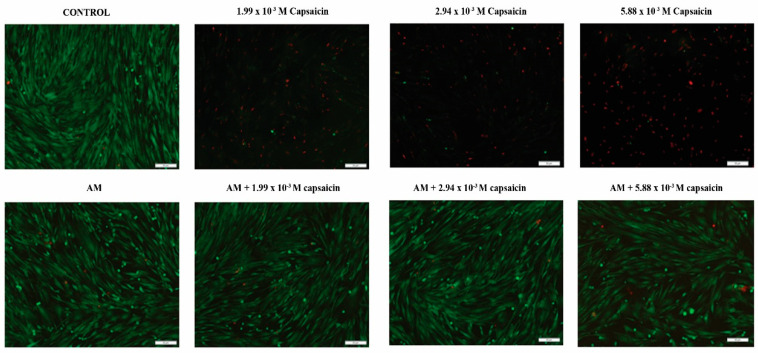
Fluorescence micrographs of live/dead stained CCD-1070Sk cells after 24 h exposure to different solutions of capsaicin and various extracts collected from simple AM or capsaicin-loaded AM (green fluorescence: live cells; red fluorescence: dead cells).

In order to obtain an overview of the potential cytotoxic effects of free and microencapsulated capsaicin on human dermal fibroblasts, cellular viability was evaluated by labelling both live and dead cells using a Live/Dead assay. The images captured by fluorescence microscopy investigation of the samples are presented in Figure 4. Our results show that all concentrations of free capsaicin dramatically alter cellular viability, since almost exclusively dead cells are scattered on the culture surface after the exposure of CCD-1070Sk cells to treatment. However, no dead cells were detected in CCD-1070Sk cell cultures exposed to 24 h-collected extracts, showing that neither simple AM nor drug-loaded AM trigger cell death in human dermal fibroblasts cells in vitro. The ratio and distribution of live cells on the cell culture surface in CCD-1070Sk cells exposed to the tested extracts for 24 h are similar to the control cell culture, showing that both bare and loaded AM lack cytotoxic potential towards human dermal fibroblasts.

The results revealed that the novel microformulation loaded with capsaicin presents no significant cytotoxicity, irrespective of the loaded concentration of capsaicin. Therefore, we selected the microformulation loaded with the highest concentration of capsaicin, 5.88 × 10^−3^ M, for further studies; in this view, extracts collected from 5.88 × 10^−3^ M capsaicin AM after 24 h were further used as cell treatments.

### 2.3. CCD-1070Sk Cell Morphology after Capsaicin-Loaded AM Treatment

**Figure 5:**
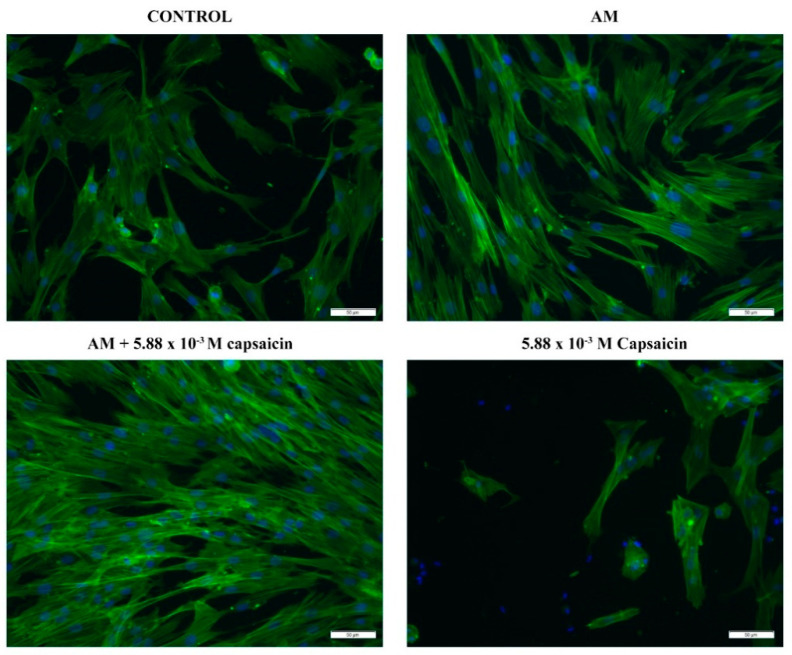
CCD-1070Sk cell morphology after 24 h of exposure to 5.88 × 10^−3^ M capsaicin, AM extract, and 5.88 × 10^−3^ M capsaicin AM extract as revealed by phalloidin staining of actin filaments (green). Cellular nuclei are stained with 2-(4-amidinophenyl)-1*H*-indole-6-carboxamidine (DAPI) (blue).

In order to investigate the potential differences in cellular morphology between human dermal cells treated with capsaicin or capsaicin-loaded AM (24 h extracts), we investigated the cytoskeleton actin filaments after FITC–phalloidin staining. As shown in Figure 5, a low number of CCD-1070Sk cells were still adhered to the culture surface after 24 h of treatment with 5.88 × 10^−3^ M capsaicin. Moreover, the few adhered cells presented with a polygon-like shape and failed to develop long actin filaments. Regarding simple or capsaicin-loaded AM treatments, it is clearly shown that none of the treatments adversely affected the global morphology of human dermal fibroblasts. After 24 h of treatment, CCD-1070Sk cells showed a typical spindle-like shape and a well-developed cytoskeleton characterized by long actin filaments. Interestingly, CCD-1070Sk cells treated with capsaicin-loaded AM presented with a better developed actin filament network compared to cells treated with pristine AM. These results suggest that microencapsulated capsaicin stimulates actin protein expression and promotes intercellular contacts.

### 2.4. Oxidative Stress Induced by Capsaicin-Loaded AM Treatment in CCD-1070Sk Cells

**Figure 6:**
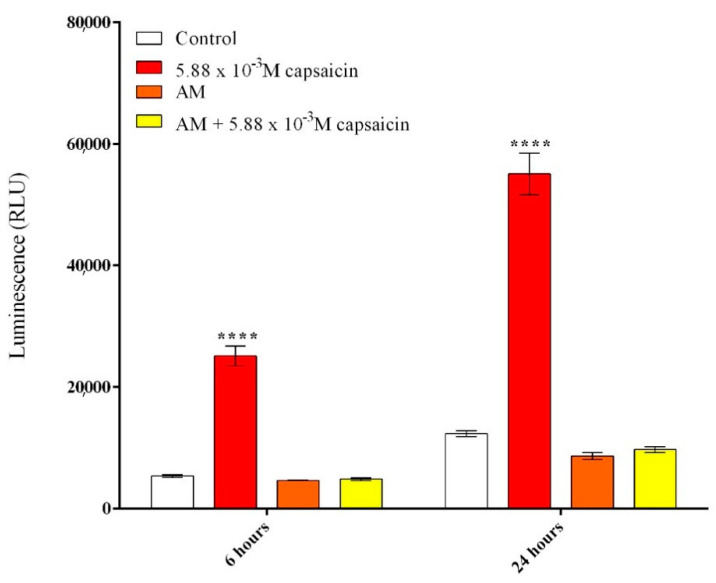
Effects of capsaicin and microencapsulated capsaicin treatment on ROS production after 6 h and 24 h of treatment in CCD-1070Sk cells as revealed by the ROS–Glo H_2_O_2_ assay. (**** *p* ≤ 0.001 treated vs. control).

In order to evaluate the potential of capsaicin-loaded AM (24 h extracts) to induce reactive oxygen species (ROS), the production of H_2_O_2_ was quantified as an indicator of oxidative stress using a ROS–Glo H_2_O_2_ assay. The obtained data are graphically represented in Figure 6 and revealed that free capsaicin induces ROS generation, as significantly enhanced levels of H_2_O_2_ were detected in treated CCD-1070Sk cell cultures compared to control cultures. Moreover, free capsaicin induced a rapid generation of hydrogen peroxide, as a significant increase in H_2_O_2_ levels was detected even after 6 h of treatment. In contrast, alginate microencapsulated capsaicin suppressed ROS production as no modifications to H_2_O_2_ levels were observed in AM and capsaicin–AM treated CCD-1070Sk cells compared to control cultures. Therefore, we conclude that alginate microcapsules and capsaicin-loaded microcapsules lack the ability to trigger H_2_O_2_ production in CCD-1070Sk cell cultures, as both treatments did not augment H_2_O_2_ levels after 6 h or 24 h of treatment.

**Figure 7:**
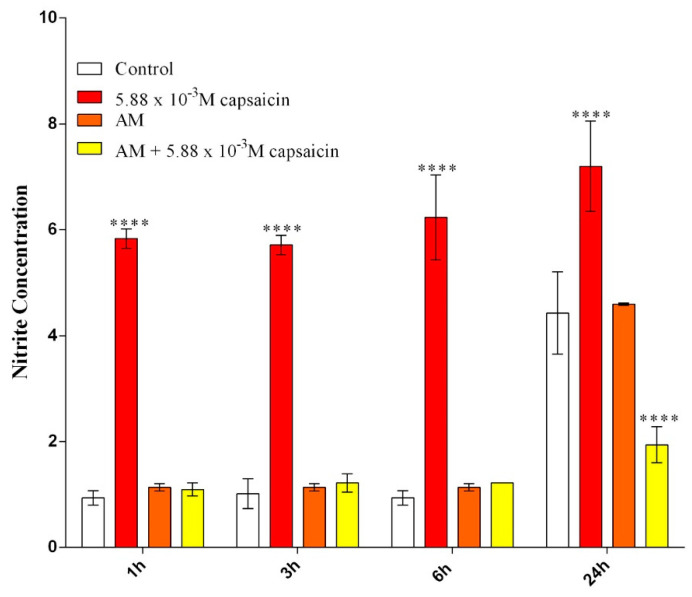
Effects of capsaicin and microencapsulated capsaicin treatment on NO production after 1 h, 3 h, 6 h, and 24 h of treatment in LPS-stimulated RAW 264.7 cells as revealed by Griess reagent assay (**** *p* ≤ 0.0001 treated vs. control).

Lipopolysaccharide (LPS) from *E. coli* was used to stimulate RAW 264.7 macrophages to produce NO. Griess reagent was used to determine NO production as an indicator of oxidative stress. The obtained data are graphically represented in Figure 7 and revealed that free capsaicin significantly increased NO generation even after 1 h of exposure compared to stimulated RAW cells. Capsaicin-loaded AM (24 h extracts), as well as unloaded AM (24 h extracts), did not alter the production of NO in the stimulated cells during the first 6 h of the experiment. However, after 24 h, capsaicin-loaded AM (24 h extracts) significantly decreased NO production compared to the control.

### 2.5. Evaluation of the Anti-Inflammatory Activity of Microencapsulated Capsaicin

**Figure 8:**
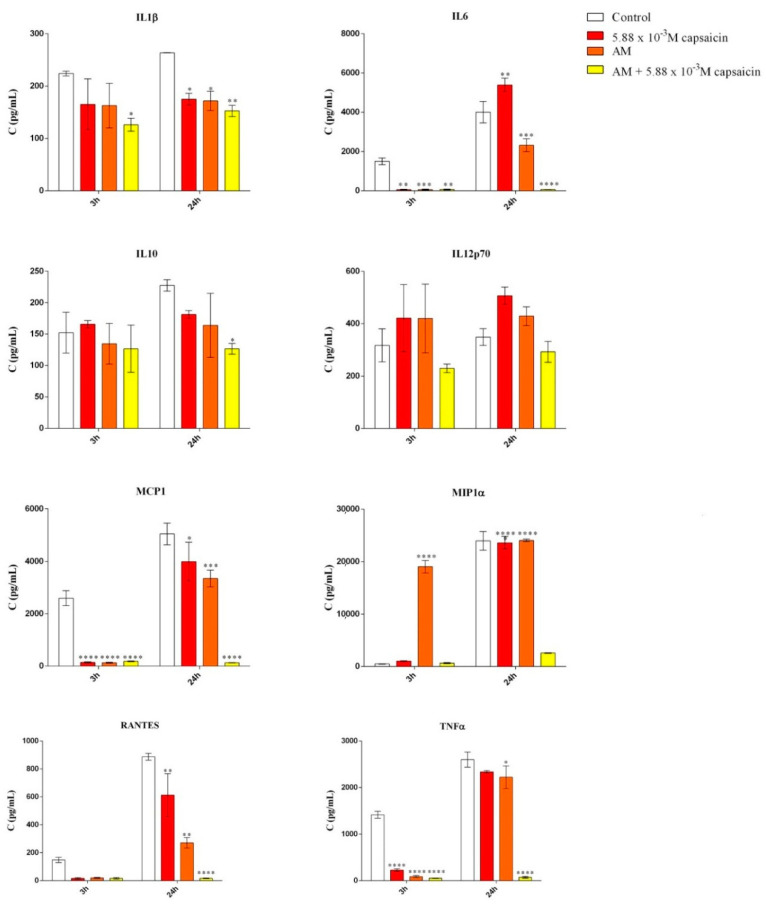
Effects of capsaicin and microencapsulated capsaicin treatment on IL-1β, IL-6, IL-10, IL-12p70, MCP-1, MIP-1α, RANTES, and TNF-α production after 3 h and 24 h of treatment in LPS-stimulated RAW 264.7 cells (* *p* ≤ 0.05 treated vs. control; ** *p* ≤ 0.01 treated vs. control; *** *p* ≤ 0.001 treated vs. control; **** *p* ≤ 0.0001 treated vs. control).

Lipopolysaccharide (LPS) from *E. coli* was used to stimulate RAW 264.7 cells to produce cytokines. The expression of IL-1β, IL-6, IL-10, IL-12p70, MCP-1, MIP-1α, RANTES, and TNF-α were determined after 3 h and 24 h of LPS stimulated RAW 264.7 cell exposure to free capsaicin and capsaicin-loaded AM (24 h extracts) using a custom Mouse Cytokine/Chemokine Magnetic Bead Panel 96-well plate assay (MCYTOMAG-70k, Merck Millipore). The results are presented in Figure 8 and show that the treatments had a significant impact, starting from 3 h of exposure for many cytokines. More specifically, the expression of IL-1β, a pro-inflammatory cytokine involved in pain [14], was significantly decreased after 3 h and 24 h of treatment with capsaicin-loaded AM compared to the untreated LPS stimulated cells. IL-6 is an inflammatory cytokine responsible for regulating a wide-range of biological pathways, including the development of acute pathological pain [15]. Our results show that the expression of IL-6 was significantly decreased after 3 h of treatment with free capsaicin, unloaded AM, and capsaicin-loaded AM extracts. However, after 24 h of treatment with free capsaicin, IL-6 levels were found to be significantly increased, while treatment with encapsulated capsaicin revealed a significand decrease in IL-6 expression. Similar patterns of expression were observed for MCP-1, MIP-1α, RANTES, and TNF-α. Raised levels of MCP-1 have been observed in patients with chronic muscle pain, while direct evidence for its role as an algogen is still lacking. MIP-1α mediates the development of neuropathic pain following peripheral nerve injury through IL-1β upregulation [16]. RANTES is a pro-inflammatory chemokine that directly interacts with opioid receptors and modifies the nociceptive reaction [17]. No significant modifications were observed with respect to the expression of IL-10 and IL-12p70 anti-inflammatory cytokines with these treatments.

## 3. Materials and Methods

### 3.1. Alginate Encapsulation of Capsaicin 

**Figure 9:**
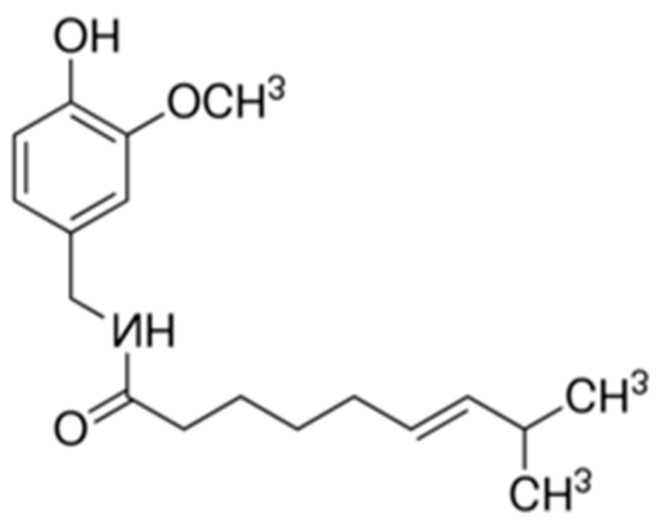
The chemical structure of capsaicin (CAS No.: 404-86-4).

Capsaicin, 8-methyl-*N*-vanillyl-6-nonenamide, is a component of a wide variety of red peppers of the genus *Capsicum*. Its chemical structure is shown in Figure 9.

Capsaicin is a hydrophobic, colorless, odorless, crystalline compound with the molecular formula C_18_H_27_NO_3_; its melting point is 62–65 °C, and its molar mass is 305.4 g/mol [4,9].

The DrugBank database is a unique bioinformatics and cheminformatics resource that combines detailed drug (i.e., chemical, pharmacological, and pharmaceutical) data with comprehensive drug target (i.e., sequence, structure, and pathway) information.

Sodium alginate (SA), with a number-averaged molecular weight of 12,000–40,000, was purchased from Sigma/Merk (Steinheim, Germany) and used as received. Calcium chloride (CaCl_2_) was supplied from Sigma/Merck (Steinheim, Germany) and was used as received. Deionized water was obtained from the lab. All reagents used in this research were obtained as analytical grade.

Alginate solution was prepared by dissolving sodium alginate in distilled water and the solution (2.5%) was stirred thoroughly. Stirring was continued after complete addition until a uniform dispersion was obtained. Alginate microcapsules loaded with capsaicin were obtained by ionotropic gelation. The microcapsules were prepared by mixing in the active component (capsaicin extract in ethanol 20% *m*/*v*) followed by ultrafiltration (calculated after filtration with CHROMAFIL O-45/15 MS filters (Machinery-Nagel GmbH, Germany)), at the concentrations presented in Table 1. The resulting homogenous bubble-free alginate dispersion was extruded using a 21 G syringe needle into the gelation medium, which was kept under stirring to improve the mechanical strength of the beads and to prevent aggregation of the formed beads. The rate of addition was 1.0 mL/min at 1100 rpm of stirring speed. The gelation medium was prepared by dispersing different concentrations of calcium chloride solution (5%).

Encapsulation evaluation has been calculated using Equation (1):

EE (%) = (P_loading_ − P_filtration_) / P_loading_ × 100 (1)

where EE = encapsulation efficiency, %; P_loading_ = amount of encapsulated capsaicin; P_filtration_ = amount of capsaicin in the ultrafiltrate.

A SPECORD M400 (Analytik Jena GmbH, Jena, Germany) and a NOVEX 100 (Novex, Arnhem, The Netherlands) were used for analysis. UV-Vis absorption spectra and the degree of sorption of capsaicin were monitored in solution with a SPECORD M400 spectrophotometer with a monochromator and double beam. Optical microscopy was performed with a NOVEX 100 microscope using the correct magnitude [18,19].

The microcapsules can be mechanically dispersed by agitation in an aqueous environment; they are sensitive to strong agitation, can be destroyed by large shear forces, and are destroyed by exposure to environments with pH values lower than 5.5 or greater than 7. The microcapsules prepared in this manner were maintained for 30 min in the gelling bath with stirring, and then filtered, washed with distilled water, and dried in an oven at 40 °C.

### 3.2. Cell Culture Models and Treatments 

The CCD-1070Sk human dermal fibroblast cell line (ATCC^®^ CRL-2091™) was used as an in vitro model for cytotoxicity investigations, as the proposed formulation is intended for skin topical application, while the RAW 264.7 mouse monocyte macrophage cell line (ATCC^®^ TIB-71™, Manassas, VA, USA) was used to model in vitro inflammatory status. Both cell lines were purchased from the American Type Culture Collection (ATCC). CCD-1070Sk and RAW 264.7 cells were cultured in Eagle’s Minimum Essential Medium (MEM) and Dulbecco’s Modified Eagle Medium (DMEM), respectively. Both media were supplemented with 10% fetal bovine serum (FBS) and 1% antibiotic–antimycotic solution (ABAM, containing 100 U/mL penicillin, 100 µg/mL streptomycin, and 0.25 µg amphotericin B). Both cell lines were sub-cultured weekly and maintained at 37 °C in a humidified air atmosphere of 5% CO_2_ all throughout this study. Media renewal was carried out every other day.

Unloaded alginate microcapsules (AM) and alginate microcapsules loaded with different concentrations of capsaicin (Table 1) were washed with phosphate-buffered saline (PBS) supplemented with 10% ABAM solution for sterilization purposes, and then immersed in complete culture media for 24 h. After 30 min, 3 h, and 24 h, media samples were collected (henceforth referred to as extracts). Collected extracts were stored at −20 °C until use. Different capsaicin solutions were freshly prepared in complete culture media and sterilized via 0.22 μm filtration, as presented in Table 1.

### 3.3. Cytotoxicity Assays

CCD-1070Sk cells were seeded in 96-well culture plates in triplicate at a final density of 2.5 × 10^4^ cells/cm^2^, or in 12-well culture plates under the same conditions for microscopy investigation. After 24 h of incubation, the culture media was discarded and replaced with the appropriate treatments (shown in Table 1). For experimental controls, the media culture was refreshed at the time of treatment. Untreated samples were used as a reference and were prepared under identical conditions for each assay.

Cell viability was investigated using the 3-(4,5-dimethilthiazol-2-il)-2,5-dipheniltetrazolium bromide (MTT) reduction assay following 24 h of exposure to treatments [19]. Briefly, the cell medium was replaced with 1 mg/mL of freshly prepared MTT solution and incubated at 37 °C for 4 h. Subsequently, the formed formazan crystals were solubilized in 2-propanol and the absorbance was read at 550 nm using a Flex Station III microplate reader (Molecular Devices, San Jose, CA, USA).

The cytotoxic potential of the screened treatments on CCD-1070Sk dermal fibroblast cells was investigated by the spectrophotometric evaluation of lactate dehydrogenase (LDH) activity in the culture media. Therefore, following 24 h of exposure to treatments, the culture medium was harvested and mixed with the components of the TOX-7 kit (LDH-Based In Vitro Toxicology Assay Kit, Steinheim, Germany) according to the manufacturer’s instructions. After 30 min incubation at room temperature in the dark, the absorbance of the samples was determined at 490 nm using a Flex Station III microplate reader (Molecular Devices).

A live/dead fluorescence assay was employed to image cells under treatment conditions. Briefly, CCD-1070Sk cells were stained with a two-color dye solution containing calcein AM (green) and ethidium bromide (red), freshly prepared according to the instructions provided by the manufacturers, in order to highlight live and dead cells at the same time. CCD-1070Sk cells were then incubated at room temperature in the dark for 20 min with the staining solution and imaged after PBS washing using an Olympus IX73 inverted fluorescence microscope. Images were captured using the CellSense imaging software (Olympus, Tokyo, Japan).

### 3.4. Cell Morphology Evaluation

The morphological changes induced by free capsaicin and microencapsulated capsaicin in CCD-1070Sk cell cultures were evaluated by the fluorescent labelling of F-actin filaments. CCD-1070Sk cells were seeded in 12-well culture plates at a final density of 2.5 × 10^4^ cells/cm^2^ and incubated for 24 h with AM, 5.88 × 10^−3^ M capsaicin-loaded AM extracts collected at 24 h, and 5.88 × 10^−3^ M capsaicin solution. After exposure, the test media was discarded and the monolayers were washed with PBS, fixed with 4% paraformaldehyde for 20 min, and permeabilized with 0.1% Triton X-100/2% bovine serum albumin for 1 h. Next, the samples were incubated for 1 h with Alexa Fluor 488–phalloidin (Thermo Fischer Scientific) at 37 °C to label the actin filaments, and nuclei were counterstained with 2 μg/mL 4′,6-diamidino-2-phenylindole dihydrochloride (DAPI; Sigma Aldrich) for 15 min. The samples were then inspected by fluorescence microscopy using an Olympus IX73 inverted microscope. Image capturing was performed using CellSense software (Olympus).

### 3.5. Reactive Oxygen Species (ROS) Assessment

Reactive oxygen species (ROS) production was measured by the ROS–Glo H_2_O_2_ assay (Promega, Madison, WI, USA), which quantifies the level of hydrogen peroxide released in the culture medium. Briefly, 9.5 × 10^4^ cells/well were seeded in a 12-well culture plate and treated with the collected extracts and different concentrations of capsaicin. For the final 6 h of treatment, H_2_O_2_ substrate was added at a final concentration of 25 μM and incubated at 37 °C in a humidified atmosphere of 5% CO_2_. After 6 h and 24 h of treatment, 100 μL of ROS–Glo Detection Solution was added, and the plate was incubated for a further 20 min at room temperature. Finally, luminescence was determined with a Flex Station III microplate reader (Molecular Devices).

### 3.6. Inflammatory Status Investigation

RAW 264.7 cells were seeded in 96-well culture plates at a final density of 2.5 × 10^4^ cells/cm^2^ in triplicate. After 24 h of incubation, the culture media was discarded and replaced with AM, 5.88 × 10^−3^ M capsaicin loaded AM extracts collected at 24 h, and 5.88 × 10^−3^ M capsaicin solution. Simultaneously, cells were stimulated with lipopolysaccharide (LPS, 10 μg/mL). For the experimental controls, the culture media was refreshed, and cells were also stimulated with LPS. After 2 h, 6 h, and 24 h, the culture medium was harvested and stored at −20 °C until use.

The collected culture media was further used to quantify the nitric oxide (NO) concentration by a method described by Griess [20] using Griess reagent (Promega). Firstly, 50 μL of culture supernatant was mixed with 50 μL sulfanilamide solution and incubated at room temperature in the dark for 20 min. After 10 min of incubation, 50 μL of *N*-1-napthylethylenediamine dihydrochloride (NED) solution was added. The optical density of the resulting solution was read at 550 nm using a Flex Station III microplate reader (Molecular Devices). The concentration of NO was extrapolated from a nitrite standard reference curve that was prepared according to the instructions provided by the manufacturer.

In order to evaluate the inflammatory status of RAW 264.7 cells after treatment, the expression of a panel of cytokines was assessed using a custom Mouse Cytokine/Chemokine Magnetic Bead Panel 96-well Plate Assay (MCYTOMAG–70k; Merck Millipore, Steinhein, Germany). Concentrations of interleukin 1β (IL-1β); interleukin 6 (IL-6); interleukin 10 (IL-10); interleukin 12p70 (IL-12p70); monocyte chemoattractant protein-1 (MCP-1); macrophage inflammatory protein 1α (MIP1α); regulated on activation, normal T cell expressed, and secreted (RANTES); and tumor necrosis factor α (TNF-α) were measured using the multiplex magnetic bead panel kit. Deposited aliquots (25 µL) of cell culture medium were incubated with anti-cytokine or anti-chemokine antibody-immobilized beads, detection antibodies, and streptavidin–phycoerythrin according to the manufacturer’s instructions. If needed, samples were adequately diluted in order to fit the linear portion of the standard curve. The plate was analyzed using a MAGPIX reader equipped with xPONENT software (Sigma/Merk, Steinheim, Germany). Standards and quality controls were assayed in duplicate as recommended by the manufacturer. The obtained data were analyzed using MILLIPLEX analysis software (Sigma/Merck, Steinheim, Germany).

## 4. Conclusions

In this study, we showed that our proposed capsaicin encapsulation system alleviates the compound’s cytotoxicity, as human dermal fibroblasts showed increased cell viability and decreased LDH activity after 24 h of treatment with capsaicin-loaded AM compared to control cells. Oxidative stress evaluation proved that the alginate microcapsules and capsaicin-loaded microcapsules were not able to trigger H_2_O_2_ production in CCD-1070Sk cell cultures. Furthermore, using a macrophage cell line for in vitro modeling of an inflammatory environment, we showed that the capsaicin-loaded AM extract significantly decreased NO production compared to the control, while treatment with the same extracts from the encapsulated capsaicin revealed a significand decrease in IL-6 expression compared to the controls. Similar patterns of expression were observed for MCP-1, MIP-1α, RANTES, and TNF-α.

## Figures and Tables

**Table 1:** Treatment regimens.

Treatment	Extracts
Alginate microcapsules (AM)	Collected after 30 min, 3 h, and 24 h
1.99 × 10^−3^ M capsaicin-loaded AM	Collected after 30 min, 3 h, and 24 h
2.94 × 10^−3^ M capsaicin-loaded AM	Collected after 30 min, 3 h, and 24 h
5.88 × 10^−3^ M capsaicin-loaded AM	Collected after 30 min, 3 h, and 24 h
1.99 × 10^−3^ M capsaicin	−
2.94 × 10^−3^ M capsaicin	−
5.88 × 10^−3^ M capsaicin	−

## Data Availability

The data presented in this study are available on request from the corresponding author.

## Sample Availability

Samples of the compounds are not available from the authors.
